# A guide to selecting high-performing antibodies for ASM (UniProt ID: P17405) for use in western blot and immunoprecipitation

**DOI:** 10.12688/f1000research.178749.1

**Published:** 2026-04-28

**Authors:** Vera Ruíz Moleón, Charles Alende, Maryam Fothouhi, Sara González Bolívar, Vincent Francis, Riham Ayoubi, Peter S. McPherson, Carl Laflamme

**Affiliations:** 1 Department of Neurology and Neurosurgery, Structural Genomics Consortium, Montreal Neurological Institute-Hospital, Montreal, Québec, Canada

**Keywords:** P17405, SMPD1, ASM, Acid sphingomyelinase, Sphingomyelin phosphodiesterase, antibody characterization, antibody validation, western blot, immunoprecipitation, immunofluorescence

## Abstract

The *SMPD1* gene encodes the acid sphingomyelinase (ASM) protein, a lysosomal enzyme essential for hydrolysing sphingomyelin into ceramide and phosphorylcholine, a key step in lipid metabolism and membrane homeostasis. Here we have characterized nine ASM commercial antibodies for western blot and immunoprecipitation using a standardized experimental protocol based on comparing read-outs in knockout cell lines and isogenic parental controls. These studies are part of a larger, collaborative initiative seeking to address antibody reproducibility issues by characterizing commercially available antibodies for human proteins and publishing the results openly as a resource for the scientific community. While the use of antibodies and protocols vary between laboratories, we encourage readers to use this report as a guide to select the most appropriate antibodies for their specific needs.

## Introduction

The sphingomyelin phosphodiesterase 1 ( *SMPD1*) gene encodes acid sphingomyelinase (ASM), a lysosomal hydrolase that converts sphingomyelin to ceramide, a bioactive lipid critical for membrane integrity and cellular signaling. ^1^ Loss-of-function mutations in *SMPD1* impair ASM activity, causing sphingomyelin accumulation and leading to Niemann-Pick disease types A and B, a lysosomal storage disorder characterized by neurodegeneration, neuroinflammation, and white matter abnormalities. ^2^ Beyond its canonical role in lysosomal degradation, ASM has been implicated in regulating membrane microdomain organization, autophagy, exosome release, and neuroimmune activation. ^3, 4^ In Parkinson’s disease, reduced ASM activity and altered ceramide metabolism are associated with increased risk and earlier onset. ^5^ Experimental models further show that ASM inhibition can modulate microglial activation, synaptic plasticity, and neuronal survival, underscoring the tight link between sphingolipid metabolism and brain homeostasis. ^6^ These findings position *SMPD1* as a molecular bridge between lysosomal function and neurodegenerative mechanisms, offering novel avenues for biomarker development and therapeutic intervention.

This research is part of a broader collaborative initiative in which academics, funders and commercial antibody manufacturers are working together to address antibody reproducibility issues by characterizing commercial antibodies for human proteins using standardized protocols, and openly sharing the data. ^7^ It consists of identifying human cell lines with adequate target protein expression and the development/contribution of equivalent knockout (KO) cell lines, followed by antibody characterization procedures using most commercially available antibodies against the corresponding protein. ^7^ Here we characterized nine commercial ASM antibodies, selected and donated by participant antibody manufacturers, for use in western blot and immunoprecipitation, enabling biochemical and cellular assessment of ASM properties and function.

The authors do not engage in result analysis or offer explicit antibody recommendations. Our primary aim is to deliver top-tier data to the scientific community, grounded in Open Science principles. This empowers experts to interpret the characterization data independently, enabling them to make informed choices regarding the most suitable antibodies for their specific experimental needs. Guidelines on how to interpret antibody characterization data found in this study are featured on the YCharOS gateway ^8^ and in Table 4 of this data note. ^7^

**Table 1.** Summary of the cell lines used.

Institution	Catalog number	RRID (Cellosaurus)	Cell line	Genotype
ATCC	HTB-14	CVCL_0022	U-87 MG	WT

**Table 2.** Summary of the ASM antibodies tested.

Company	Catalog number	Lot number	RRID (Antibody Registry)	Clonality	Clone ID	Host	Concentration (μg/μL)	Vendors recommended applications
Abcam	ab272729 **	1070539–3	AB_3105948	Recombinant mono	EPR23090–181	Rabbit	0.50	Wb
Abcam	ab277243 **	1050795-5	AB_3105956	Recombinant mono	EPR23090–192	Rabbit	1.04	other
Abcam	ab277244 **	105	AB_3101951	Recombinant mono	EPR23090–4	Rabbit	0.99	other
Abcam	ab315810 **	1076948-7	AB_3101783	Recombinant mono	EPR28613–49	Rabbit	0.49	Wb, IF
Bio-Techne (Novus biologicals)	NBP2–45889 *	F003	AB_3097708	Monoclonal	OTI3H7	Mouse	1.00	Wb
Bio-Techne (R&D systems)	MAB5348 *	CEIM0121101	AB_10640504	Monoclonal	563418	Mouse	0.50	IP
Cell signaling technology	3687	1	AB_1904135	Polyclonal	-	Rabbit	0.06	Wb
Proteintech	14609–1-AP	00124670	AB_2878068	Polyclonal	-	Rabbit	0.50	Wb
Thermo fisher scientific	MA5–26615 *	ZF4351577	AB_2723927	Monoclonal	OTI3H7	Mouse	1.00	Wb

Wb = western blot; IF = immunofluorescence; IP = immunoprecipitation,

** = recombinant antibody,

* = monoclonal antibody

**Table 3.** Table of secondary antibodies used.

Company	Secondary antibody	Catalog number	RRID (Antibody Registry)	Clonality	Concentration (μg/μL)	Working concentration (μg/mL)
Proteintech	HRP-Goat Anti-Rabbit Antibody (H + L)	RGAR001	AB_3073505	Recombinant polyclonal	1.0	0.05
Proteintech	HRP-Goat Anti-Mouse Antibody (H + L)	RGAM001	AB_3068333	Recombinant polyclonal	1.0	0.5
Abcam	Anti-mouse IgG for IP (HRP)	ab131368	AB_2895114	Monoclonal	1.0	2.0

**Table 4.** Illustrations to assess antibody performance in all western blot and immunoprecipitation.

Western blot	Immunoprecipitation
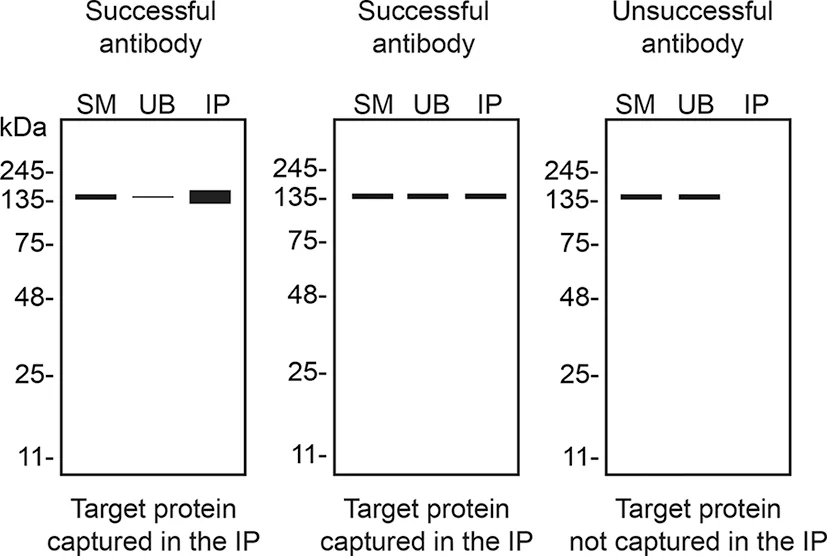	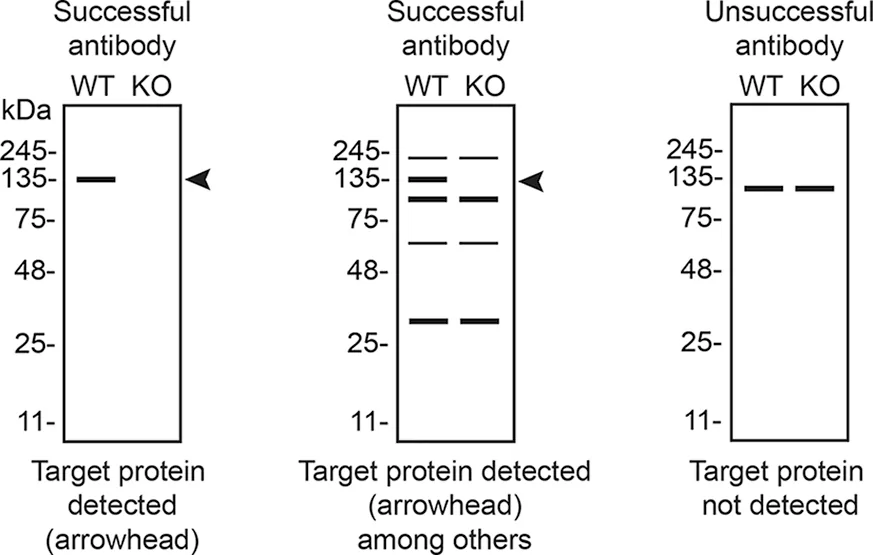

This table has been reproduced with permission from Ayoubi et al., Elife, 2023 ^12^

## Results and discussion

Our standard protocol involves comparing readouts from wild type (WT) and KO cell line. ^9^ In the absence of a commercially available KO in a cell line background that expresses adequate levels of ASM, siRNA technology is employed to knockdown (KD) the target gene. ^10, 11^ To determine which cell line demonstrates high expression of ASM and thus be appropriate for KD, the first step is to identify a cell line that expresses sufficient levels of a given protein to generate a measurable signal using antibodies. To this end, we examined the DepMap (Cancer Dependency Map Portal, RRID: SCR_017655) transcriptomics database to identify cell lines that express the target at levels greater than 2.5 log _2_ (transcripts per million “TPM” + 1), which we have found to be a suitable cut-off. ^12^ We selected the U-87 MG cell line that expresses the *SMPD1* gene at 6.3 log _2_ TPM + 1. A non-targeting control siRNA pool was used to treat U-87 MG control (ctrl) cells, while *SMPD1* was KD using a pool of siRNA targeting this gene.

According to the UniProt database, ASM is predominantly secreted. To detect secreted ASM, all nine antibodies were first screened by western blot with U-87 MG ctrl and *SMPD1* KD proteins from culture medium ran on SDS-PAGE, transferred onto nitrocellulose membranes, and then probed with the nine ASM antibodies in parallel ( Figure 1A). Additionally, selected antibodies were then tested for intracellular ASM detection in protein extracts from U-87 MG control and *SMPD1* knockdown cells ( Figure 1B). Although ASM was detectable in cell lysates, the signal required longer exposure times and higher protein loading compared to medium samples, indicating that most ASM is secreted. Based on these findings, the antibodies were not evaluated by immunofluorescence in this study.

**Figure 1.**
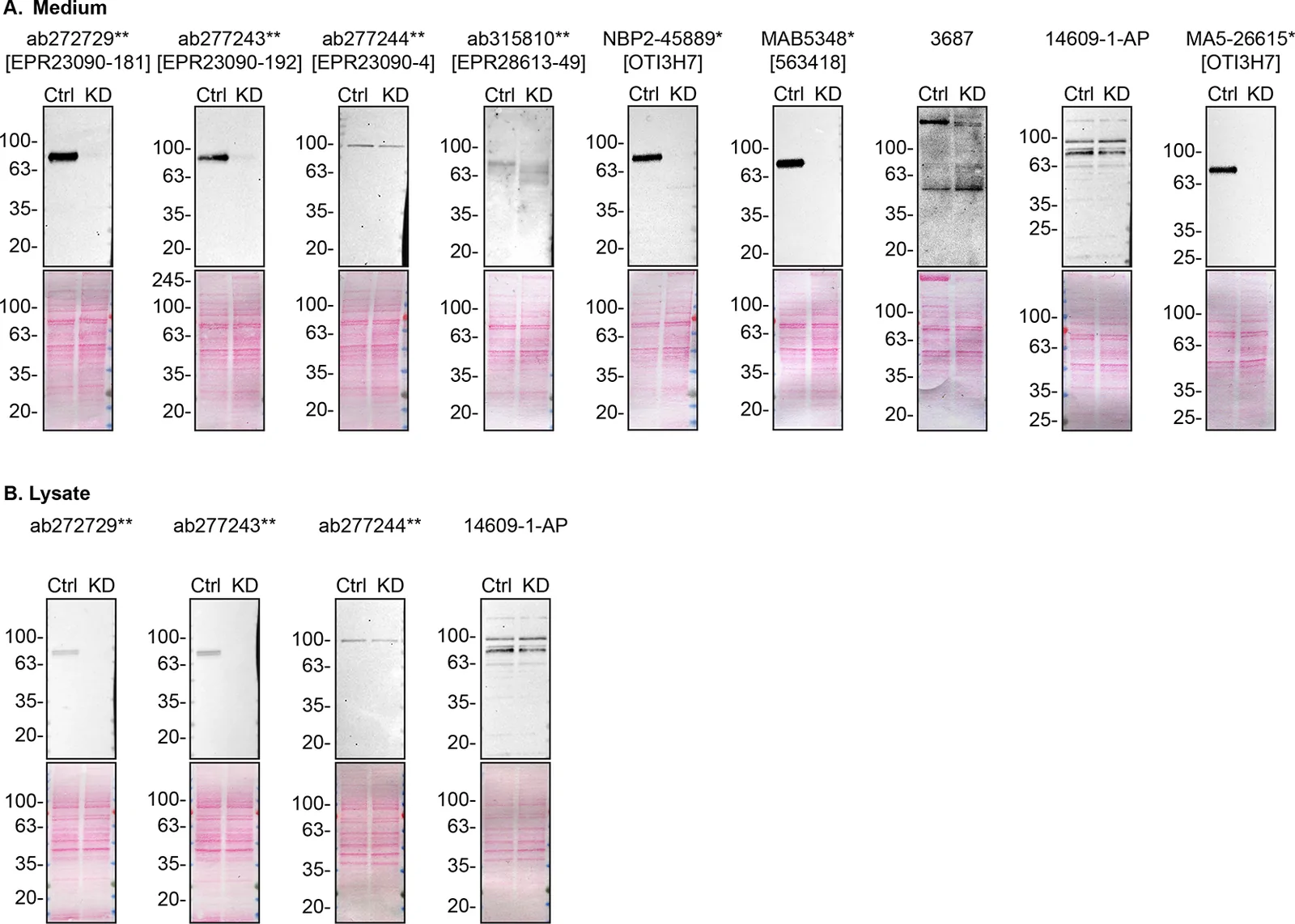
ASM antibody screening by western blot. **A)** Culture media from U-87 MG ctrl and *SMPD1* KD were collected, and 10 μg of protein were processed for western blot with the indicated ASM antibodies. The Ponceau stained transfers of each blot are presented to show equal loading of ctrl and KD samples and protein transfer efficiency from the acrylamide gels to the nitrocellulose membrane. Antibody dilutions were chosen according to the recommendations of the antibody supplier. Antibody dilutions used: ab272729** at 1/1000, ab277243** at1/1000, ab277244** at 1/1000, ab315810 at 1/200, NBP2–45889* at 1/500, MAB5348* at 1/200, 3687 at 1/200, 14609–1-AP at 1/500 and MA5–26615* at 1/500. **B)** Lysates of U-87 MG ctrl and *SMPD1* KD were prepared, and 30 μg of protein were processed for western blot with the selected ASM antibodies used at the same dilution as in A). Predicted band size: 69.9 kDa. ** = recombinant antibody, * = monoclonal antibody.

We then assessed the capability of the nine antibodies to capture ASM from U-87 MG culture medium using the immunoprecipitation technique, followed by western blot analysis. For the immunoblot step, a specific ASM antibody identified previously (refer to Figure 1) was selected. Equal amounts of the starting material (SM) and the unbound fractions (UB), as well as the whole immunoprecipitate (IP) eluates were separated by SDS-PAGE ( Figure 2).

**Figure 2.**
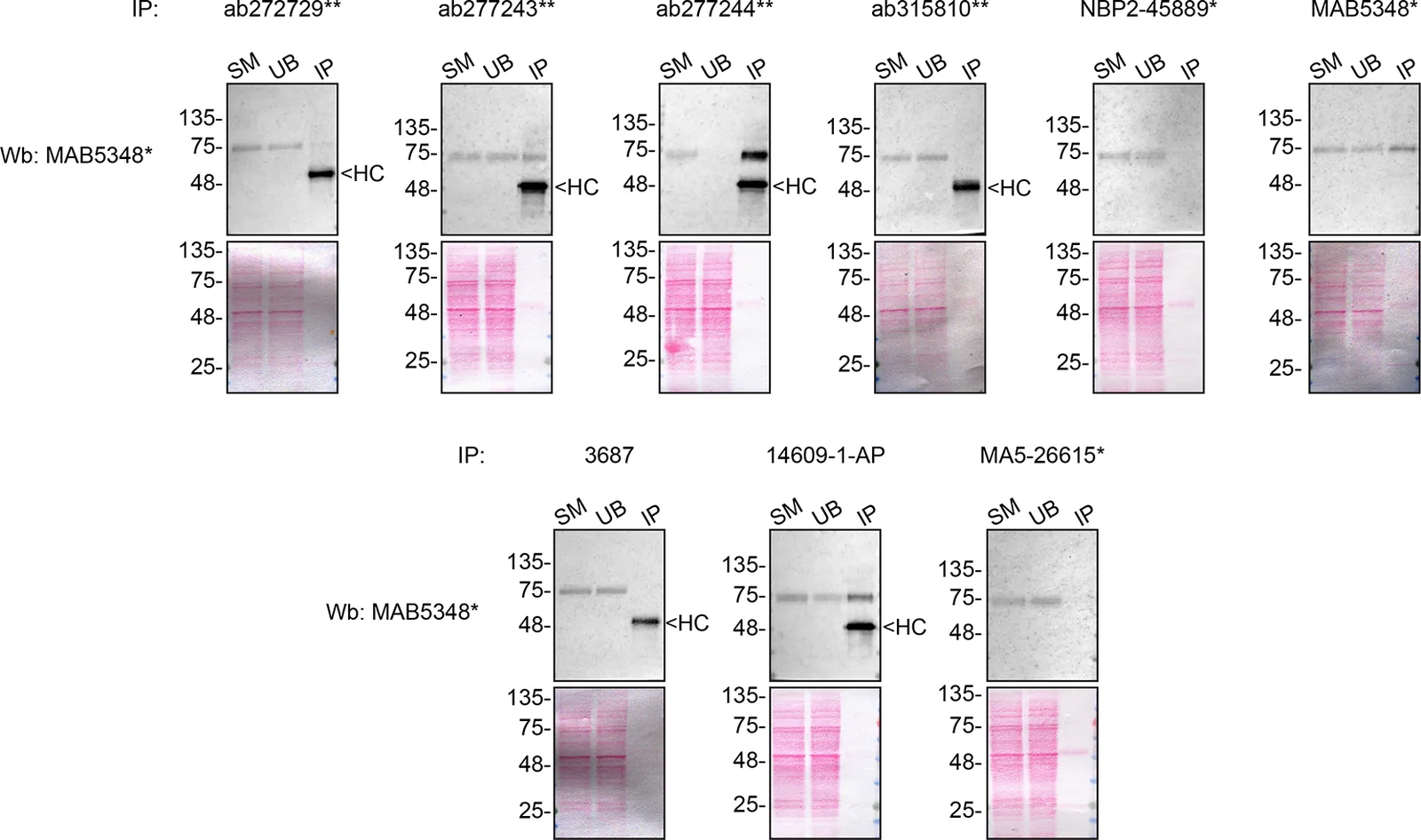
ASM antibody screening by immunoprecipitation on culture medium. Culture medium was collected from U-87 MG WT, and immunoprecipitation was performed for 1 h using 0.3 mg of protein and 2.0 μg of the indicated ASM antibodies pre-coupled to Dynabeads protein A or protein G. Samples were washed and processed for western blot with the anti-ASM MAB5348* diluted at 1/500. The Ponceau stained transfers of each blot are shown. SM = 4% starting material; UB = 4% unbound fraction; IP = immunoprecipitate, HC = antibody heavy chain. ** = recombinant antibody, * = monoclonal antibody.

In conclusion, we have screened nine ASM commercial antibodies by western blot and immunoprecipitation by comparing the signal produced by the antibodies in human U-87 MG ctrl and *SMPD1* KD cells. To assist users in interpreting antibody performanyce, Table 4 outlines various scenarios in which antibodies may perform in these applications. ^12^ Several high-quality and renewable antibodies that successfully detect ASM were identified in both applications. Researchers who wish to study ASM in a different species are encouraged to select high-quality antibodies, based on the results of this study, and investigate the predicted species reactivity of the manufacturer before extending their research.

## Limitations

Inherent limitations are associated with the antibody characterization platform used in this study. Firstly, the YCharOS project focuses on renewable (recombinant and monoclonal) antibodies and does not test all commercially available ASM antibodies. YCharOS partners provide approximately 80% of all renewable antibodies, but some top-cited polyclonal antibodies may not be available through these partners.

Secondly, the YCharOS effort employs a non-biased approach that is agnostic to the protein for which antibodies have been characterized. The aim is to provide objective data on antibody performance without preconceived notions about how antibodies should perform or the molecular weight that should be observed in western blot. As the authors are not experts in ASM, only a brief overview of the protein’s function and its relevance in disease is provided. ASM experts are invited to analyze and interpret observed banding patterns in western blots and subcellular localization in immunofluorescence.

Thirdly, YCharOS experiments are not performed in replicates primarily due to the use of multiple antibodies targeting various epitopes. Once a specific antibody is identified, it validates the protein expression of the intended target in the selected cell line, confirms the lack of protein expression in the KO cell line and supports conclusions regarding the specificity of the other antibodies. Moreover, the same antibody clones are donated by 2–3 manufacturers (cross-licensed antibodies), effectively serving as replicates and enabling the validation of test reproducibility. All experiments are performed using master mixes, and meticulous attention is paid to sample preparation and experimental execution. In IF, the use of two different concentrations serves to evaluate antibody specificity and can aid in assessing assay reliability. In instances where antibodies yield no signal, a repeat experiment is conducted following titration. Additionally, our independent data is performed subsequently to the antibody manufacturers internal validation process, therefore making our characterization process a repeat.

Lastly, as comprehensive and standardized procedures are respected, any conclusions remain confined to the experimental conditions and cell line used for this study. The use of a single cell type for evaluating antibody performance poses as a limitation, as factors such as target protein abundance significantly impact results. Additionally, the use of cancer cell lines containing gene mutations poses a potential challenge, as these mutations may be within the epitope coding sequence or other regions of the gene responsible for the intended target. Such alterations can impact the binding affinity of antibodies. This represents an inherent limitation of any approach that employs cancer cell lines.

## Method

The standardized protocols used to carry out this KO cell line-based antibody characterization platform was established and approved by a collaborative group of academics, industry researchers and antibody manufacturers. The detailed materials and step-by-step protocols used to characterize antibodies in western blot, immunoprecipitation and immunofluorescence are openly available on Protocols.io ( protocols.io/view/a-consensus-platform-for-antibody-characterization ). ^7^ Brief descriptions of the experimental setup used to carry out this study can be found below.

To knockdown *SPMD1*, U-87 MG cells were treated with the SMARTPool ON-TARGETplus Human *SMPD1* siRNA from Horizon Discovery (cat. number L-006676-00-0005) for five days. U-87 MG control cells were treated with the ON-TARGETplus Non-targeting Control Pool (Horizon Discovery, cat. number. D-001810-10). Lipofectamine RNAiMAX (Thermo Fisher Scientific, cat. number 13778030) was used to transfect the siRNA following the manufacturer’s protocol.

### Cell lines and antibodies

The cell lines, primary and secondary antibodies used in this study are listed in Table 1, 2, and 3, respectively. To ensure consistency with manufacturer recommendations and account for proprietary formulations (where antibody concentrations are not disclosed), antibody usage is reported as dilution ratios rather than absolute concentrations. To facilitate proper citation and unambiguous identification, all cell lines and antibodies are referenced with their corresponding Research Resource Identifiers (RRIDs). ^13, 14^ All cell lines used in this study were regularly tested for mycoplasma contamination and were confirmed to be mycoplasma-free.

### Antibody screening by western blot

U-87 MG ctrl and *SMPD1* KD cells were washed 3x with PBS 1x and starved for ~36 hrs. Culture media were collected and centrifuged for 10 min at 500 x *g* to eliminate cells and larger contaminants, then for 10 min at 4500 x *g* to eliminate smaller contaminants. Culture media were then concentrated by centrifuging at 4000 x *g* for 30 min using the Amicon Ultra-15 Centrifugal Filter Units with a membrane NMWL of 10kDa (MilliporeSigma cat. number UFC901024). Culture media were finally supplemented with 1x protease inhibitor cocktail mix (MilliporeSigma, cat. number P8340). BLUelf prestained protein ladder (GeneDireX, cat. number PM008-0500) was used. For lysate preparation, cells were collected in RIPA buffer (25mM Tris-HCl pH 7.6, 150mM NaCl, 1% NP-40, 1% sodium deoxycholate, 0.1% SDS) (Thermo Fisher Scientific, cat. number 89901) supplemented with 1x protease inhibitor cocktail mix. Lysates were sonicated briefly and incubated 30 min on ice. Lysates were spun at ~110,000 *x g* for 15 min at 4°C and equal protein aliquots of the supernatants were analyzed by SDS-PAGE and western blot.

Western blots were performed with precast midi 4-20% Tris-Glycine polyacrylamide gels (Thermo Fisher Scientific, cat. number WXP42012BOX) ran with Tris/Glycine/SDS buffer (Bio-Rad, cat. number 1610772), loaded in Laemmli loading sample buffer (Thermo Fisher Scientific, cat. number AAJ61337AD) and transferred on nitrocellulose membranes. Proteins on the blots were visualized with Ponceau S staining (Thermo Fisher Scientific, cat. number BP103-10) which is scanned to show together with individual western blot. Blots were blocked with 5% milk for 1 hr, and antibodies were incubated O/N at 4°C with 5% milk in TBS with 0,1% Tween 20 (TBST) (Cell Signalling Technology, cat. number 9997). Following three washes with TBST, the peroxidase conjugated secondary antibody was incubated at a dilution of ~0.2 μg/ml in TBST with 5% milk for 1 hr at room temperature followed by three washes with TBST. Membranes were incubated with Pierce ECL (Thermo Fisher Scientific, cat. number 32106) or Clarity Western ECL Substrate (Bio-Rad, cat. number 1705061) prior to detection with the iBright™ CL1500 Imaging System (Thermo Fisher Scientific, cat. number A44240).

### Antibody screening by immunoprecipitation

Antibody-bead conjugates were prepared by adding 2 μg of antibody to 500 μl of Pierce IP Lysis Buffer (25 mM Tris-HCl pH 7.4, 150 mM NaCl, 1 mM EDTA, 1% NP-40 and 5% glycerol) from Thermo Fisher Scientific, cat. number 87788 in a microcentrifuge tube, together with 30 μl of protein A- (for rabbit antibodies) or protein G- (for mouse antibodies) (Thermo Fisher Scientific, cat. number 10002D and 10004D, respectively). Tubes were rocked for ~1 h at 4°C followed by two washes to remove unbound antibodies.

Culture media from U-87 MG WT were collected as described in the western section above. 0.75 mL aliquots at 0.4 mg/mL of culture medium were incubated with an antibody-bead conjugate for ~1 h at 4°C. The unbound fractions were collected, and beads were subsequently washed three times with 1.0 mL of IP buffer and processed for SDS-PAGE and western blot on precast midi 4–20% Tris-Glycine polyacrylamide gels.

## Data Availability

Zenodo: Dataset for the ASM antibody screening study < https://doi.org/10.5281/zenodo.17407436 >. Data are available under the terms of the Creative Commons Attribution 4.0 International license (CC-BY 4.0).

## References

[1] Kornhuber J Rhein C Muller CP : Secretory sphingomyelinase in health and disease. *Biol. Chem.* 2015;396(6–7):707–736. 25803076 10.1515/hsz-2015-0109

[2] Jones I He X Katouzian F : Characterization of common SMPD1 mutations causing types A and B Niemann-Pick disease and generation of mutation-specific mouse models. *Mol. Genet. Metab.* 2008;95(3):152–162. 18815062 10.1016/j.ymgme.2008.08.004 PMC2621017

[3] Huang D Li G Bhat OM : Exosome biogenesis and lysosome function determine podocyte exosome release and glomerular inflammatory response during hyperhomocysteinemia. *Am. J. Pathol.* 2022;192(1):43–55. 34717894 10.1016/j.ajpath.2021.10.005 PMC8759037

[4] Ermini L Farrell A Alahari S : Ceramide-induced lysosomal biogenesis and exocytosis in early-onset preeclampsia promotes exosomal release of smpd1 causing endothelial dysfunction. *Front. Cell Dev. Biol.* 2021;9:652651. 34017832 10.3389/fcell.2021.652651 PMC8130675

[5] Gan-Or Z Orr-Urtreger A Alcalay RN : The emerging role of SMPD1 mutations in Parkinson's disease: Implications for future studies. *Parkinsonism Relat. Disord.* 2015;21(10):1294–1295. 26320887 10.1016/j.parkreldis.2015.08.018

[6] Bi J Khan A Tang J : Targeting glioblastoma signaling and metabolism with a re-purposed brain-penetrant drug. *Cell Rep.* 2021;37(5):109957. 34731610 10.1016/j.celrep.2021.109957 PMC8856626

[7] Ayoubi R Ryan J Gonzalez Bolivar S : A consensus platform for antibody characterization. *Nat. Protoc.* 2024;20:1509–1545. 10.1038/s41596-024-01095-8 PMC13054593 39690206

[8] Biddle MS Virk HS : YCharOS open antibody characterisation data: Lessons learned and progress made. *F1000Res.* 2023;12:1344. 10.12688/f1000research.141719.1 PMC10579855 37854875

[9] Laflamme C McKeever PM Kumar R : Implementation of an antibody characterization procedure and application to the major ALS/FTD disease gene C9ORF72. *elife.* 2019;8. 10.7554/eLife.48363 PMC6794092 31612854

[10] Fire A Xu S Montgomery MK : Potent and specific genetic interference by double-stranded RNA in Caenorhabditis elegans. *Nature.* 1998;391(6669):806–811. 10.1038/35888 9486653

[11] Dana H Chalbatani GM Mahmoodzadeh H : Molecular mechanisms and biological functions of siRNA. *Int. J. Biomed. Sci.* 2017;13(2):48–57. PMC5542916 28824341

[12] Ayoubi R Ryan J Biddle MS : Scaling of an antibody validation procedure enables quantification of antibody performance in major research applications. *elife.* 2023;12:12. 10.7554/eLife.91645 PMC10666931 37995198

[13] Bandrowski A Pairish M Eckmann P : The antibody registry: ten years of registering antibodies. *Nucleic Acids Res.* 2023;51(D1):D358–D367. 36370112 10.1093/nar/gkac927 PMC9825422

[14] Bairoch A : The cellosaurus, a cell-line knowledge resource. *J. Biomol. Tech.* 2018;29(2):25–38. 29805321 10.7171/jbt.18-2902-002 PMC5945021

