# Supplementary material for: A guide to selecting high-performing antibodies for ASM (UniProt ID: P17405) for use in western blot and immunoprecipitation
Source: F1000Res. 2026 Apr 28;15:632. [Version 1] doi: 10.12688/f1000research.178749.1 (PMC13717167; doi:10.12688/f1000research.178749.1)
Supplement: Peer review history

# Reviewer report: Reviewer response for version 1

**DOI:** 10.5256/f1000research.197174.r529596

**Published:** 2026-10-28

**Authors:** Deborah Moshinsky

**Affiliations:** 1 Institute for Protein Innovation, Boston, Massachusetts, USA

Acid sphingomyelinase (ASM) protein is a lysosomal enzyme essential for hydrolysing sphingomyelin into ceramide and phosphorylcholine, a key step in lipid metabolism and membrane homeostasis. The authors characterized nine commercial ASM antibodies for use in western blot and immunoprecipitation as part of a larger antibody characterization initiative, using standardized protocols.

 The authors found that some antibodies worked in Western blot, meaning a band of the appropriate size was seen in wild type cells but not in cells where the target was knocked down.  Others successfully immunoprecipitated ASM from cell lysate.  This study should serve as an appropriate guide for researchers seeking antibodies for ASM that are likely to work in their application.

 The methodologies are clear and well described.  Researchers should be able to reproduce the findings based on this report.

 In Figure 4, the panel describing "unsuccessful antibody Western Blot" says target protein not detected.  This is not necessarily correct.  I suggest rephrasing to say "target protein detection unclear," because it could be detecting the target protein among others.  The conclusion of unsuccessful antibody remains.

Are sufficient details of methods and materials provided to allow replication by others?

Yes

Is the rationale for creating the dataset(s) clearly described?

Yes

Are the datasets clearly presented in a useable and accessible format?

Yes

Are the protocols appropriate and is the work technically sound?

Yes

Reviewer Expertise:

Antibody Characterization and Validation

I confirm that I have read this submission and believe that I have an appropriate level of expertise to confirm that it is of an acceptable scientific standard.

# Reviewer report: Reviewer response for version 1

**DOI:** 10.5256/f1000research.197174.r494415

**Published:** 2026-07-02

**Authors:** Karen J Bowman

**Affiliations:** 1 University of Leicester, Leicester, England, UK

The study involved characterisation of nine commercial antibodies, from six different suppliers, for the acid sphingomyelinase (ASM) protein for use in western blot, and immunoprecipitation. The study formed part of the wider YCharOS collaboration to characterise commercial antibodies for human proteins using standardised protocols with the intention of improving antibody reproducibility issues. As a Data Note, it allows the direct comparison of the performance of commercially available antibodies to ASM protein to aid scientists in choosing the most appropriate antibody for biochemical and cellular assessment of ASM properties and function. For example, in the study of, lysosomal degradation, the regulation of membrane microdomain organization, autophagy, exosome release, and neuroimmune activation. This comparison would be of particular use in the fields of lipid metabolism and membrane homeostasis.

 A committee of industry and academic representatives have endorsed the platform used, and the protocols used are consistent with those in general use. The platform consisted of identification of a human cell line suitable for antibody characterisation studies i.e., with adequate levels of the protein to generate a measurable signal. They found that the commercially available U-87 MG cell line expresses the sphingomyelin phosphodiesterase 1 ( *SMPD1*) gene (which encodes ASM) at a level above the average range for the cancer cells analysed, and therefore, was a logical choice for their study. Knockdown of the target gene using siRNA technology, with a pool of siRNAs, was used to provide appropriate negative control cells. This was used as there was no commercial KO in a cell line background that expresses adequate levels of ASM available. The final step was a series of antibody characterisation procedures, limited to the most common research uses of antibodies. The ASM protein is predominantly secreted into the cell culture medium rather than located within the cells, therefore, cell culture medium was collected for analysis, in addition to the usual cell lysates. Due to the low levels of ASM detected in cell lysates, immunofluorescence studies were not undertaken.

 The interpretation of the results is left to the reader, with the study providing unbiased guidance. However, it gives some indication of the optimum dilutions of commercial antibodies. Limitations of the study are clearly stated.

 The study is a useful starting point when choosing the most appropriate antibody for study of ASM by western blot or immunoprecipitation.

 There are a few corrections that could be made as follows,

- In figure legend 1A, a space is required between at and 1 in “ab277243** at1/1000”.
- In the limitations section, the sentence “In IF, the use of two different concentrations serves to evaluate antibody specificity and can aid in assessing assay reliability.” should be removed as no IF was done in this study.
- In the methods, antibody screening by western blot section, paragraph 2, the buffer the 5 % milk is made up in should be stated in the sentence “Blots were blocked with 5% milk for 1 hr,” (presumably it is TBST).

Are sufficient details of methods and materials provided to allow replication by others?

Yes

Is the rationale for creating the dataset(s) clearly described?

Yes

Are the datasets clearly presented in a useable and accessible format?

Yes

Are the protocols appropriate and is the work technically sound?

Yes

Reviewer Expertise:

Drug discovery, development and diagnostics.

I confirm that I have read this submission and believe that I have an appropriate level of expertise to confirm that it is of an acceptable scientific standard.
